# Complete Genome Sequence of Bovine Viral Diarrhea Virus 2 Japanese Reference and Vaccine Strain KZ-91CP

**DOI:** 10.1128/genomeA.01573-14

**Published:** 2015-02-12

**Authors:** Asuka Sato, Ken-ichiro Kameyama, Makoto Nagai, Kentaro Tateishi, Keitaro Ohmori, Reiko Todaka, Kazuhiko Katayama, Tetsuya Mizutani, Makoto Yamakawa, Junsuke Shirai

**Affiliations:** a Cooperative Department of Veterinary Medicine, Faculty of Agriculture, Tokyo University of Agriculture and Technology, Fuchu, Tokyo, Japan; b Research and Education Center for Prevention of Global Infectious Diseases of Animals, Tokyo University of Agriculture and Technology, Fuchu, Tokyo, Japan; c National Institute of Animal Health, National Agriculture and Food Research Organization, Tsukuba, Ibaraki, Japan; d Department of Virology II, National Institute of Infectious Diseases, Musashimurayama, Tokyo, Japan

## Abstract

In this study, we report the complete genome sequence of the bovine viral diarrhea virus 2 Japanese reference strain KZ-91CP. The complete genome comprises 12,654 nucleotides and one open reading frame with 4,020 amino acids. A 369-nucleotide-long insertion encoding the chaperone protein DnaJ is found in the nonstructural 2 (NS2) coding region.

## GENOME ANNOUNCEMENT

Bovine viral diarrhea virus 2 (BVDV-2) is a member of the genus *Pestivirus* within the family *Flaviviridae*, together with the proposed species BVDV-1, classical swine fever virus, and border disease virus. BVDV-2 was first reported during an outbreak of bovine severe acute hemorrhagic syndrome in North America and Canada in the early 1990s (1, 2). Thereafter, BVDV-2 isolates were identified using samples collected worldwide from cattle with clinical signs similar to those accompanying BVDV-1 infection. KZ-91CP was isolated in 1991 from a Holstein cow age 30 months with mucosal disease and was recognized as the first BVDV-2 strain in Japan (3). This strain is used as an inactivated vaccine and a reference virus for laboratory diagnosis in Japan. Recently in Japan, of the two BVDV types (BVDV-1 and BVDV-2), BVDV-2a comprises 16% of all strains of BVDV (4); however, the complete genome sequences of Japanese BVDV-2a strains, including that of KZ-91CP, remained undetermined. This background prompted us to sequence the whole genome of KZ-91CP. In this study, we report the complete genome sequence of Japanese BVDV-2 reference strain KZ-91CP.

Total viral RNA was extracted from infected primary bovine fetal muscle cells using Isogen-LS (Nippon Gene, Toyama, Japan) and normalized to 50 ng using a Qubit 2.0 fluorometer (Invitrogen, Carlsbad, CA). Library construction for deep sequencing was performed using the NEBNext Ultra RNA library prep kit for Illumina version 2.0 (New England BioLabs, Ipswich, MA), according to the manufacturer’s guidelines. After assessing the library quality and quantity on a bioanalyzer (Agilent Technologies, Santa Clara, CA) and Qubit 2.0 fluorometer, sequencing was conducted on a MiSeq benchtop sequencer. Sequence data analysis was conducted using the MiSeq reporter program to generate FastQ-formatted sequence data. The contig was assembled from the obtained sequence reads with the *de novo* assembly command in CLC Genomics Workbench 6.0 (CLC bio, Aarhus, Denmark). To confirm the sequences of the contig, Sanger sequencing of four overlapping reverse transcription-PCR (RT-PCR) products was performed. The nucleotide sequences of the 5′-untranslated region (UTR) and 3′-UTR termini were determined using the rapid amplification of cDNA ends method (5, 6).

The complete genome of KZ-91CP comprises 12,654 nucleotides (nt), including a 386-nt-long 5′ UTR, a 205-nt-long 3′ UTR, and a large open reading frame that encompasses 4,020 amino acids (aa). The nonstructural 2 (NS2) coding region contains a 369-nt-long insertion (at amino acid position 1532). BVDVs are classified into two biotypes, cytopathogenic (cp) and noncytopathogenic (ncp), based on their effect on cultured cells (7). Many cp viruses carry host cell-derived insertions and/or duplicated rearranged viral genomic sequences (8, 9). KZ-91CP exhibits a cp phenotype and possesses an insertion encoding the chaperone protein DnaJ (10), which is most frequently observed in the vicinity of position A (aa position 1535) in BVDV-2 (11). KZ-91CP has higher homologies at the amino acid level with recent American and German BVDV-2 strains (98% to 99%) than with the BVDV-2 prototype strain 890 (2a) (91.3%).

### Nucleotide sequence accession number. 

The complete genome sequence of BVDV strain KZ-91CP has been deposited in DDBJ/ENA/GenBank under the accession no. LC006970.
